# Recombinant *Ganoderma lucidum* Immunomodulatory Protein Improves the Treatment for Chemotherapy-Induced Neutropenia

**DOI:** 10.3389/fphar.2020.00956

**Published:** 2020-06-26

**Authors:** Xinhua Lei, Chenyang Zhi, Wei Huang, Xilin Sun, Weiyang Gao, Xue Yin, Xin Zhang, Chongyang Liang, Haoran Zhang, Fei Sun

**Affiliations:** ^1^ School of Pharmaceutical Sciences, Jilin University, Changchun, China; ^2^ Anorectal, Changchun University of Traditional Chinese Medicine Affiliated Hospital, Changchun, China; ^3^ Department of New Drug Development, Changchun Intellicrown Pharmaceutical Co. LTD, Changchun, China; ^4^ Neurosurgery, Gaoyou People's Hospital, Yangzhou, China

**Keywords:** hematopoietic stem cells, neutropenia, rLZ-8, granulocyte-colony stimulating factor, colony-stimulating factor 1 receptor

## Abstract

*Ganoderma lucidum*, also known as LINGZHI, has a long tradition of use in folk medicine of the Far East, which is documented in the oldest Chinese pharmacopoeia, declaring it a superior medicine. LINGZHI-8 (LZ-8) is an immunoregulatory fungal protein isolated from the fruiting body of *Ganoderma lucidum*. Neutropenia is a condition with an abnormally low levels of neutrophils in the blood, which is caused by numerous medical conditions or medications, such as chemotherapy. The current study demonstrated that recombinant LZ-8 (rLZ-8) from Pichia promoted the differentiation of bone marrow hematopoietic stem cells (HSCs) into granulocytes in a neutropenia mouse model induced by cyclophosphamide. Also, it regulated the CXCR4-SDF1 axis to promote the mobilization of HSCs and the release of neutrophils from the bone marrow to peripheral blood. Binding of rLZ-8 to the colony stimulating factor 1 receptor (CSF1R) promotes the differentiation of HSCs into primitive CFU colonies. These results suggested that rLZ-8 has a potential effect in the treatment of chemotherapy-induced neutropenia.

## Introduction

*Ganoderma lucidum* is a major medicinal fungus widely used in traditional Chinese medicine. *Ganoderma lucidum* immunomodulatory protein (LZ-8) is a fungal protein isolated from the fruiting body of the fungus. It consists of 110 amino acids, and the immunoglobulin-like structure formed a biologically active non-covalently linked homodimer with a role in the immunomodulatory functions as well as in the inhibition of tumor growth (Kohsuke et al., 1991; Yeh et al., 2010). Reportedly, LZ-8 induced the activation and maturation of human dendritic cells *via* the nuclear factor kappa-light-chain-enhancer of activated B cells (NF-κB) and mitogen-activated protein kinase (MAPK) pathways (Lin et al., 2009). It also served as a potential adjuvant for the development of DNA vaccines for human cancers owing to its stimulatory activity on dendritic cells (Chu et al., 2011). Furthermore, the recombinant LZ-8 (rLZ-8) induced the degradation of epidermal growth factor receptor (EGFR) in a clathrin-mediated, endocytosis-dependent, and c-Cbl ubiquitination-dependent manner, which in turn, inhibited proliferation and promoted apoptosis of lung cancer cells (Lin et al., 2017). Currently, the class of chemicals or proteins that exhibit the anti-cancer effects, as well as maintain the homeostasis of immune cells are not identified. The results of another study demonstrated that FIP-gts promotes the recovery of white blood cells (WBCs). FIP-fve and FIP-gts significantly improved the chemotherapy-induced myelosuppression and intestinal mucosal damage, diminished the risk of bone metastasis risk and restored bone microenvironment (Ou et al., 2015). Our previous studies demonstrated that rLZ-8 significantly increased the number of WBCs in a mouse model of cyclophosphamide-induced leukopenia (Zhou et al., 2013). However, the effector cell types and the underlying proliferation mechanisms have not yet been identified. The present study will follow the previous work.

Neutrophils are the most abundant leukocytes in the circulation of human body and the first line of defense against the infection. These groups of cells participated in the activation, regulation, and effector functions of innate and adaptive immune cells (Mantovani et al., 2011). Neutropenia is the most common complication of myelosuppressive anti-cancer therapy. Cytotoxic chemotherapy inhibited the hematopoietic system, impaired the body's self-protection mechanisms, and limited the tolerable dose of chemotherapeutics. The patients were posed with a high risk of bacterial and fungal infections (Crawford et al., 2004). In order to facilitate the immune functions and the relatively short survival time, a continuous production of neutrophils in the bone marrow was required, which was a highly regulated and energy-consuming process (Amulic et al., 2012). Granulocyte colony-stimulating factor (G-CSF) is a vital regulator of this process (Richards et al., 2003). It promotes the differentiation of myeloid progenitor cells into granulocyte lineage by increasing the expression of the transcription factors PU.1 and C/EBPβ (Zhu and Emerson, 2002; Hirai et al., 2006). On the other hand, it induced stem cell mobilization by regulating the CXCR4-SDF1 pathway and promoted the migration of mature neutrophils from the bone marrow to the blood to satisfy the acute requirements of the body suffering from infection and hematopoietic suppression (Petit et al., 2002; Summers et al., 2010). However, the number of neutrophils increased sharply but diminished subsequently during the course of G-CSF treatment, which is an unstable process. Moreover, only a few therapeutic agents are available for the treatment of neutropenia.

To understand the mechanism underlying the treatment of chemotherapy-induced neutropenia, we established a stable mouse model to determine the effects of rLZ-8 on increasing the number of neutrophils in the peripheral blood and bone marrow. Also, the mechanisms promoting the proliferation and differentiation of neutrophils were compared to those of G-CSF. The proliferation pathway and release process of neutrophils under the action of rLZ-8 was studied *in vitro* and *in vivo* in order to determine whether the recombinant protein can be used as a potential agent for the treatment of neutropenia.

## Materials and Methods

### Preparation of rLZ-8

The expression of the LZ-8 plasmid and the purification protocol of the protein was similar to that described previously (Liang et al., 2012). The LZ-8 gene was cloned into the pGAPZαA vector (Thermo Fisher Scientific, Waltham, MA, USA). The rLZ-8 was transformed into Pichia pastoris X33, according to the manufacturer's instructions. At OD600 = 6.0, the cells were transferred into a BioFlo310 Bioreactor (New Brunswick Scientific, Enfield, CT, USA) with pre-addition of 3.5 L BSM medium, 8 ml biotin, and 12 ml PTM1 and cultured at 29°C, 800 rpm, and 20% DO-STAT; also, glycerol was added continuously during the process to ensure the expression of rLZ-8. Samples were withdrawn every 6 h to measure the expression of rLZ-8 by polyacrylamide gel electrophoresis (SDS-PAGE). After 66 h of induction, the supernatant was collected by centrifugation at 4°C and 12000×g for 10 min. The rLZ-8 protein was purified using the SP Sepharose XL preparative column (GE Healthcare, Uppsala, Sweden). The endotoxin levels of rLZ-8 were determined by limulus amebocyte lysate assay. The column volume: 1.5 L; mobile phase A: pH 3.5 and 50 mM NaAC-HAc; mobile phase B: 1.5 M NaCl was solubilized in phase A. Phase B was eluted with a 0%-70% gradient of 10 column volumes at a flow rate of 35 ml/min. The peak fraction from ÄKTA purification was subjected to HPLC detection using a molecular sieve column (Shimadzu Corporation, Kyoto, Japan); mobile phase: pH 6.8, 0.02 M NaH_2_PO_4_-Na_2_HPO_4_, and flow rate 0.6 ml/min.

### Animals

BALB/c and C57BL/6J mice were purchased from Charles River Laboratories and maintained at conventional temperature, humidity, and light conditions. 8-10-week-old BALB/c male mice were used for the pharmacodynamic test, and 6-week-old C57BL/6J female mice were used for the enrichment of hematopoietic stem cells (HSCs), respectively. All the animal experiments were performed in Jilin University Animal Center under sterile condition in an SPF facility in accordance with the animal welfare laws and the regulations of the Association for Assessment and Accreditation of Laboratory Animal Care (AAALAC).

### Animal Study Design

(1) A total of 210 BALB/c mice were divided into three groups: normal, single-administration model, and multiple-administrations model. Cyclophosphamide was administered at 230 mg/kg per mouse for 1 day intraperitoneally in the single-administration model group, and at 50 mg/kg for 3 days intraperitoneally in the multiple-administrations model group. Mice in the normal group received an equivalent volume of normal saline solution. WBC counts were determined before and after cyclophosphamide or normal saline injection. (2) 250 BALB/c mice were divided into five groups: normal, model, subcutaneous, intravenous, and non-model-based. Routes and dose of medication administration are described in **Supplementary Figure 1**. WBC counts were determined before and after processing. (3) 540 BALB/c mice were divided into six groups: normal, model, G-CSF, rLZ-8 low-dose, rLZ-8 medium-dose, and rLZ-8 high-dose. Routes and dose of medication administration are described in **Supplementary Figure 1**. Peripheral whole blood collected at different time-points was used to detect WBCs and neutrophils by automated hematology analyzer and flow cytometry, respectively. Bone marrow cells were collected to detect neutrophils, CXCR4^+^ cells, HSCs or hematopoietic progenitor cells by flow cytometry. Bone marrow supernatant fluid was collected and was used for the detection SDF-1 by Wes.

### Blood Cell Count

Fresh blood samples of the mice were withdrawn from the posterior orbital venous plexus in a polypropylene tube containing EDTA. The whole blood count was enumerated using a Sysmex XT-2000iv hematology analyzer (Sysmex Corporation, Kobe, Japan).

### Extraction of HSCs

Bone marrow cells were obtained from the bone marrow cavity of the femur and tibia in the mice using pre-cooled phosphate buffered solution (PBS) under sterile conditions. The red blood cells (RBCs) were lysed (BD Science, San Jose, CA, USA), and a single cell suspension was prepared using FACSmax (Amsbio, Abingdon, UK). A 200-mesh filter was used to exclude the cell pellet and residue, and the cells were preserved in the RebSep Buffer (Stem Cell, Vancouver, BC, Canada) after centrifugation (4°C at 1200 rpm for 5 min), washing, and counting. According to the operation manual, the mouse progenitor-enriched antibody-coated magnetic beads and Sca1 magnetic beads (Stem Cell, Vancouver, BC, Canada) were used to negatively remove the Lineage^+^Sca1^+^ cells. Subsequently, the cKit magnetic beads (Stem Cell, Vancouver, BC, Canada) were used to positively enrich the cKit^+^ cells that, were used for the quantification of colony forming units (CFUs) and immunofluorescence assays after assessing the purity of the Lineage^-^cKit^+^Sca1^-^ cells by flow cytometry.

### Flow Cytometry Analysis

Whole bone marrow was isolated and stained on ice with various antibody cocktails to identify each progenitor compartment. PE-CD11b and FITC-Ly6G (BD Science, San Jose, CA, USA) were used to detect the neutrophil populations, while PE-CD184 (BD Science, San Jose, CA, USA) was used to detect the CXCR4^+^ cell populations. Biotin-CD4, Biotin-CD8a, Biotin Mouse Lineage Panel, PE-CD135, V450-CD127, APC-CD117, PE-Cy7 Streptavidin (BD Science, San Jose, CA, USA), PE-Cy5.5 Sca1 (Abcam, Cambridge, UK), and AF700-CD16/32 (eBioscience, San Diego, CA, USA) were used to detect the HSCs and their differentiated cell populations. All the labeled cells were evaluated using an Aria II flow cytometer (BD Science, San Jose, CA, USA) or FC500 flow cytometer (Beckman, Brea, CA, USA), and the data were processed using FlowJo software (BD Science, San Jose, CA, USA). For detailed procedure, see **Supplementary Figure 2**.

### Quantitative Analysis of CFU Colonies

The colony formation ability of the cells was assessed based on the proliferation on the methylcellulose semi-solid medium, according to the manufacturer's protocol. An equivalent of 0.4 ml single cell suspension, containing 1×10^4^ HSCs/ml was mixed with 4 ml MethoCult™ medium (Stem cell, Vancouver, BC, Canada) to reach a volume of 1:10 to maintain the appropriate viscosity of the medium in order to ensure the optimal growth environment for the colonies. A 3-ml screw syringe and a 16-gauge blunt needle were used to subculture 1.1 ml of the cell-containing medium in a 35 mm culture dish. Subsequently, G-CSF or rLZ-8 was added, and the mixture incubated at 37°C, 5% CO_2_, and humidity ≥95% for 9 days. The type and number of colonies were recorded using an optical microscope. The hematopoietic colonies were identified as follows: colonies containing red blood cells and bone marrow cells (CFU-GEMM), myeloid colonies with pure granulocyte colonies (CFU-G), and colonies containing granulosa cells and monocytes (CFU-GM). The data were expressed as the number of CFU/1000 Lineage^-^cKit^+^Sca1^-^ cells.

### Western Blot

Bone marrow extracellular fluid was obtained by rinsing each femur with 1 ml of serum-free pre-cooled PBS and centrifugation at 1200 rpm for 5 min. The resulting bone marrow supernatant from each group was used to detect the amount of SDF-1 by capillary electrophoresis based Protein Simple Wes System. The 13-Capillary Cartridge (Protein Simple, San Jose, CA, USA) was chosen. Samples were denatured by incubation with 0.1 X sample buffer (Protein Simple, San Jose, CA, USA) at 37°C for 20 min. SDF-1 (Abcam, Cambridge, UK) primary antibody (1:50) was incubated with the sample, followed by secondary antibody. GAPDH was used as a loading control and run in another 13-capillary cartridge; the protein amount loaded was the same as that of SDF-1. Data were processed using Compass software (Protein Simple, San Jose, CA, USA).

### Immunofluorescence Imaging

HSCs were incubated with fluorescently labeled rLZ-8 in a 6-well plate containing DMEM medium (10% fetal bovine serum) for 30 min. Then, the cells were counted and inoculated on to the dried and ultraviolet-treated coverslips coated with polyethyleneimine at a density of 0.5×10^5^/cm^2^, followed by PBST (PBS with 0.1% Tween 20) washes. Subsequently, the cells were fixed with 4% paraformaldehyde for 10 min at room temperature and permeabilized with PBS containing 0.5% saponin for 10 min. Then, the non-specific antibody binding was blocked by incubation with PBST containing 1% BSA and 22.52 mg/ml glycine for 30 min. Next, the cells were incubated with primary antibodies G-CSFR, CSF1R, GM-CSFRα, and GM-CSFRβ (Abcam, Cambridge, UK), respectively, in a humid chamber at 4°C overnight, followed by fluorescent-labeled secondary antibody in 1% BSA for 1 h in the dark. Finally, after washing with PBS, the cells on the coverslip were sealed on the glass slide to avoid drying and preserved at -20°C in the dark. The samples were observed under the Structured Illumination Microscope (SIM, DeltaVision OMX, GE Healthcare, Uppsala, Sweden).

### Statistical Analysis

Data are expressed as mean ± standard deviation. The means were compared by Mann-Whitney U test. The calculations were performed using GraphPad Prism 8 (GraphPad Software San Diego, CA, USA) and the reported *P*-values were bilateral. Statistical significance was defined as *P* < 0.05.

## Results

### rLZ-8 With High Purity Was Obtained After Fermentation and Purification

**Figure 1:**
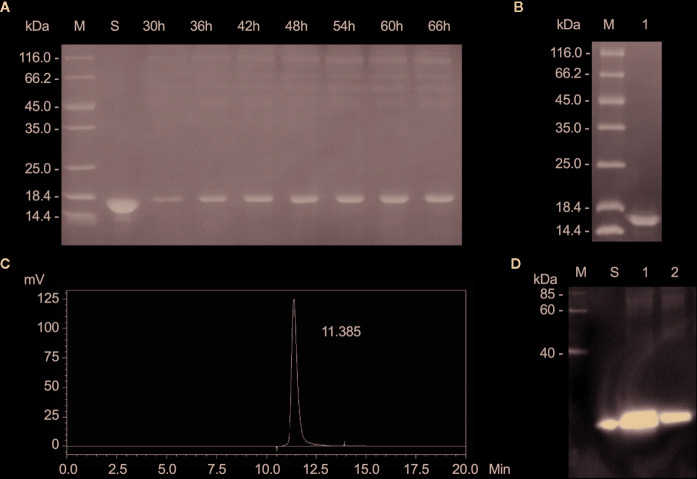
Relatively high purity of rLZ-8 protein was obtained after fermentation and purification process. **(A)** After 66 h fermentation, the position of rLZ-8 protein band was detected by SDS-PAGE in the supernatant, M: Marker, S: rLZ-8 protein standard, Lane 3-9: 30-66 h fermentation broth supernatants. **(B)** The purified rLZ-8 protein was detected by SDS-PAGE, M: Marker, Lane 1: purified rLZ-8 protein. **(C)** The purity was detected by HPLC. The only highest peak appeared at 11.385 min, and the purity was 99.89%. **(D)** Purified rLZ-8 protein and rLZ-8 protein standard was detected using Western blotting analysis, M: Marker, S: rLZ-8 protein standard, Lane 1: purified rLZ-8 protein sample 1, Lane 2: purified rLZ-8 protein sample 2.

After fermentation, the protein supernatant collected at different time points was resolved by SDS-PAGE (**Figure 1A**). Compared to the negative control, the supernatant displayed an additional band of approximately 17 kDa, its expression increases with time and heteroproteins were detected in the range of 35-116kDa. The purified sample was also detected by SDS-PAGE. The only band was detected at approximately 17kDa and no other proteins were detected in the sample (**Figure 1B**). Additionally, a large amount of rLZ-8 was accumulated in the medium with mass similar to that of the native LZ-8 protein and slightly larger than the theoretical molecular weight calculated based on the amino acid sequences. The purity of the isolated protein was 99.89% as assessed by HPLC (**Figure 1C**), and the level of endotoxin was <1.0 EU/μg (Data not shown). The purified rLZ-8 protein was detected by Western blot (**Figure 1D**). The above results demonstrated that the high purity protein fulfils the requirements of *in vivo* experiments.

### rLZ-8 Increases the Number of Peripheral Blood WBCs

**Figure 2:**
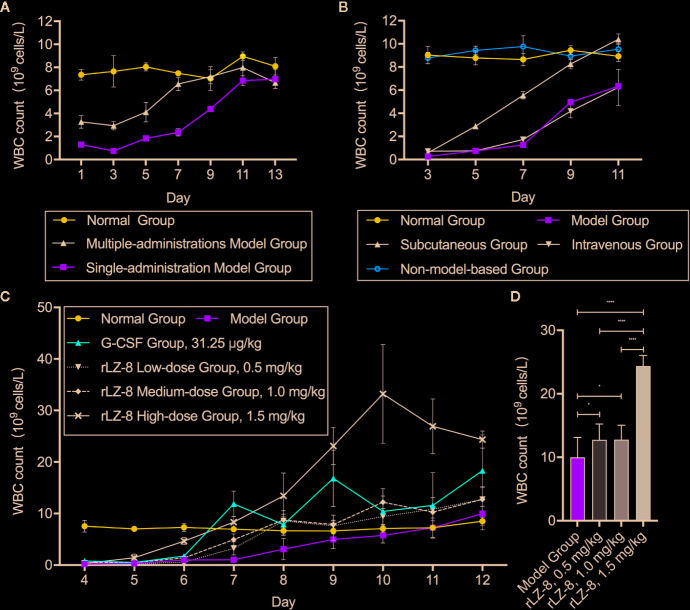
rLZ-8 increases the count of peripheral blood WBCs in a mouse model of neutropenia. **(A)** Comparison of the modeling method in mice treated by single or multiple administrations of cyclophosphamide. Single-administration model group: CY (230 mg/kg, i.p.), once a day, for 1 day, Multiple-administrations model group: CY (50 mg/kg, i.p.), once a day, for 3 days, N=10. **(B)** Effects of different rLZ-8 administration routes on the peripheral blood WBC counts in mice, routes and dose of medication administration are described in **Supplementary Figure 1**, N = 10. **(C)** Comparison of the effects of different rLZ-8 doses on the peripheral blood WBC counts in mice 3 days after model establishment, routes and dose of medication administration are described in **Supplementary Figure 1**, N = 10. **(D)** Comparison of the increase in the WBC counts on D13 in the model mice treated by different doses of rLZ-8, N=10.

To determine whether rLZ-8 increases the number of peripheral blood leukocytes in mice with neutropenia *in vivo*, we compared the stability of the model between a single intraperitoneal injection (230 mg/kg) and 3 consecutive days of intraperitoneal injection (50 mg/kg) of cyclophosphamide. We stipulated that the start time of the experiment is day 0 (D0) (**Supplementary Figure 1**). The peripheral blood routine examination showed that the number of peripheral blood WBC counts decreased to the standard count of neutropenia (0.755 ± 0.047×10^9^ cells/L) in the single administration model on D3 and returned to the baseline level (6.845 ± 0.430×10^9^ cells/L) of the normal group (7.805 ± 0.915×10^9^ cells/L) on D11, which met the experimental requirements (**Figure 2A**). Subsequently, the effects of tail vein administration and subcutaneous administration methods on increasing the number of WBCs were compared. During the 11-day administration period, the subcutaneous group showed a tendency to increase the number of WBCs on D7 (5.552 ± 0.325×10^9^ cells/L); however, no significant difference was observed between intravenous group and model group at each timepoints (*P* > 0.05). Furthermore, no significant change was detected in the number of WBCs in the non-model-based group in which the healthy mice were injected with rLZ-8 alone (**Figure 2B**).

After determining the modeling and drug administration methods, we tested the therapeutic effect of rLZ-8 on neutropenia. Three doses (0.5, 1.0, and 1.5 mg/kg) of rLZ-8 were administered subcutaneously from days 3–11 for 9 consecutive days. G-CSF (31.25 μg/kg) served as the positive treatment group. Also, the placebo group was set up. The peripheral blood WBCs were enumerated to evaluate the therapeutic effects of each group. Compared to the model group, the differences in the peripheral blood WBC counts in all the treatment groups were statistically significant during D7-12 (*P* < 0.05). In the G-CSF group, the count reached 11.877 ± 2.484×10^9^ cells/L on D7, which was almost 12-fold of that in the model group. In the rLZ-8 0.5, 1.0, and 1.5 mg/kg groups, the WBC count reached 8.592 ± 0.788×10^9^ cells/L on D8, 8.744 ± 1.785×10^9^ cells/L on D8, and 8.336 ± 1.119×10^9^ cells/L on D7, similar to that of the normal group. Moreover, the therapeutic peak was achieved on D9 in the G-CSF group (16.833 ± 5.490×10^9^ cells/L), and that in the rLZ-8 low-dose and medium-dose group was achieved on D12 (12.762 ± 2.487×10^9^ cells/L and 12.768 ± 2.292×10^9^ cells/L, respectively). Furthermore, the WBC count reached 33.224 ± 9.598×10^9^ cells/L on D10 in the high-dose rLZ-8 group, which was 3.2-fold of the peak value in the G-CSF group. It reached to 4.7-fold of that in the model group on D10 (**Figure 2C**). In addition, the effect of rLZ-8 on increasing the number of WBCs was significantly correlated with the dose of injection. After the therapeutic effect was observed, the daily WBC count in the high-dose rLZ-8 group was higher than that in the rLZ-8 low-dose and medium-dose groups. At the end of the experiment (D13), compared to the model group, the three doses of rLZ-8 treatment group showed an increase in the WBC count, while the high-dose group was still significantly different from the low-dose and medium-dose groups. However, no significant differences were observed between the low-dose and the medium-dose groups (*P* > 0.05) (**Figure 2D**). Subsequently, the dose used in the rLZ-8 treatment group was 1.5 mg/ml unless otherwise specified. These results indicated that rLZ-8 can increase the number of peripheral blood WBCs in mice with neutropenia and can achieve same or better therapeutic effects as compared to the recommended injection dosage of G-CSF.

### rLZ-8 Increases the Count of Neutrophils in Peripheral Blood and Bone Marrow

**Figure 3:**
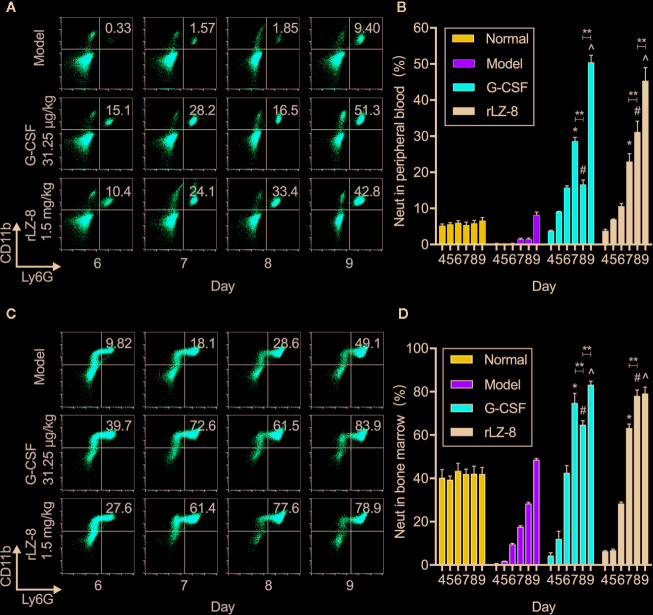
rLZ-8 increases the number of CD11b^+^Ly6G^+^ cells in peripheral blood and bone marrow in the mouse model of neutropenia. **(A)** FCM detected the number of neutrophils in the peripheral blood of mice. Normal: Vehicle, Model: Vehicle, G-CSF: 31.25 μg/kg, rLZ-8: 1.5 mg/kg. **(B)** Analysis of peripheral blood neutrophil ratio in different treatment groups, *P < 0.05 vs. Model D7, ^#^ *P* < 0.05 vs. Model D8, ^*P* < 0.05 vs. Model D9, N = 10. **(C)** FCM detected the number of neutrophils in mouse bone marrow. Normal: Vehicle, Model: Vehicle, G-CSF: 31.25 μg/kg, rLZ-8: 1.5 mg/kg. **(D)** Analysis of bone marrow neutrophil ratio in different treatment groups, *P < 0.05 vs. Model D7, ^#^ *P* < 0.05 vs. Model D8, ^*P* < 0.05 vs. Model D9, **P < 0.01, N = 10.

In order to confirm that the main component of leukocytes in peripheral blood was neutrophils under the action of rLZ-8, we evaluated the changes in the number of neutrophils during treatment in the mice from the normal, model, G-CSF, and rLZ-8 groups by flow cytometry (**Figure 3A**). The results showed that the proportion of CD11b^+^Ly6G^+^ cells in the peripheral blood of G-CSF and rLZ-8 treatment groups was altered significantly compared to the model group during D7–9 (*P* < 0.05), and that the G-CSF group showed an increasing trend, followed by a decrease and then an increase. On the other hand, the rLZ-8 group showed a trend of continuous increase, which was in agreement with the results of the routine blood WBCs test. The rLZ-8 group could achieve the therapeutic effect identical to that of the G-CSF group. On experimental D7, the percentage of peripheral blood neutrophils in the G-CSF group reached 28.660 ± 1.001%, and that in the rLZ-8 group reached 22.964 ± 2.186%, which was 19- and 15-fold, respectively as compared to that in the model group. On experimental D9, the proportion of neutrophils in the G-CSF group and the rLZ-8 group was 50.480 ± 1.901% and 45.360 ± 3.629%, respectively, indicating that the neutrophils were increased in peripheral blood after treatment with rLZ-8 (**Figure 3B**).

In order to verify that neutrophils were derived from the differentiation of bone marrow cells under the action of rLZ-8 because of the increase in the number of cells caused by the decrease in cyclophosphamide myelosuppression, we also assessed the content of neutrophils in the bone marrow by flow cytometry (**Figure 3C**). The data demonstrated that the intramedullary granulocytes in the G-CSF group began to recover on D5 (12.074 ± 3.545%); this phenomenon was observed on D6 in the rLZ-8 group (28.380 ± 0.687%). Notably, the proportion of intramedullary neutrophils on D8 in the G-CSF group (64.780 ± 1.862% was decreased as compared to that on D7 (74.900 ± 4.375%), followed by an increase on D9 (83.260 ± 1.610%). However, the neutrophil count was continuously increased during the experimental D7–9 in the rLZ-8 group. This phenomenon was consistent with the results of flow cytometry on peripheral blood. The reduction in the number of intramedullary neutrophils on D8 of the G-CSF group was a direct cause of reduction in the peripheral blood neutrophils (**Figure 3D**). Another non-negligible result was that the intramedullary neutrophils in the model group were also in a state of recovery, which might be attributed to the weakened myelosuppressive effect of cyclophosphamide. Strikingly, a specific role of cyclophosphamide was noted in promoting the growth of neutrophils. The above results indicated that the increased levels of neutrophils in the peripheral blood were derived from bone marrow cells under the action of rLZ-8 or G-CSF.

### rLZ-8 Promoted the Differentiation of HSCs Into Granulocyte Progenitor Cells in the Bone Marrow

**Figure 4:**
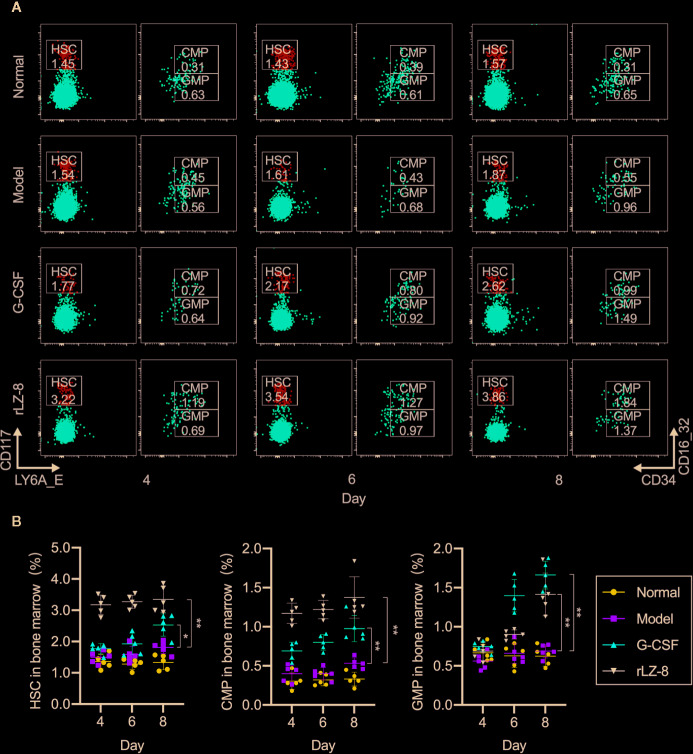
*In vivo* experiments showed that rLZ-8 improves the self-renewal of HSCs in the bone marrow and differentiation into granulocyte progenitor cells in the mouse model of neutropenia. **(A)** FCM detected the HSC/CMP/GMP cell population in the bone marrow of mice in different treatment groups on days 4/6/8. Normal: Vehicle, Model: Vehicle, G-CSF: 31.25 μg/kg, rLZ-8: 1.5 mg/kg. **(B)** Analysis of ratio of HSC/CMP/GMP cell populations in the bone marrow in different treatment groups, *P < 0.05 vs. Model D8, **P < 0.01 vs. Model D8 N = 5.

Neutrophils in the bone marrow were derived from HSCs differentiated into myeloid progenitor cells (CMPs) and downstream granulocyte and monocyte progenitor cells (GMPs). In order to test whether the increase in the number of neutrophils was the result of rLZ-8 acting on HSCs, we used flow cytometry to evaluate the changes in the three intramedullary cell populations, HSCs, CMPs, and GMPs, on experimental D4, D6, and D8. The proportion of the three cell populations in the normal group did not alter significantly (**Figure 4A**). The proportion of HSC populations increased in the G-CSF group and rLZ-8 group [2.528 ± 0.355% (1.4-fold) and 3.350 ± 0.416% (1.85-fold), respectively] as compared to the model group (1.812 ± 0.198%) on D8. Both G-CSF and rLZ-8 groups were higher than the normal group (1.334 ± 0.238%) (**Figure 4B**). In addition, the proportion of GMP cells in the G-CSF group was higher than that in the rLZ-8 group on D8, while the proportion of CMP cells in the two groups was opposite. It was speculated that rLZ-8 might act on primitive HSCs to stimulate their differentiation into CMPs.

**Figure 5:**
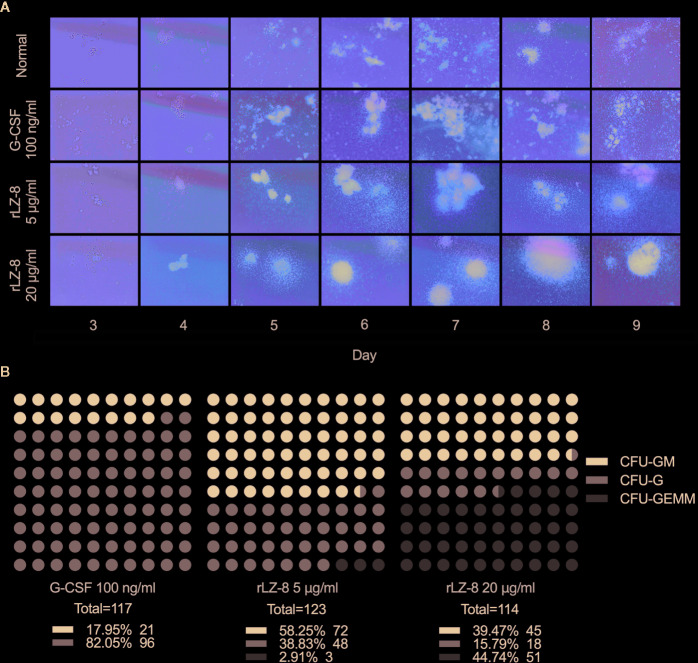
*In vitro* experiments showed that rLZ-8 promotes the differentiation of HSCs into granulocyte progenitor cells. **(A)** CFU colony formation assay was used to detect the differentiation status of hematopoietic stem cells in different administration groups. Normal: Vehicle, G-CSF: 100 ng/ml, rLZ-8 Low-dose: 5 μg/ml, rLZ-8 High-dose: 20 μg/ml. **(B)** Analysis of the types and numbers of colony differentiation in each experimental group on D9 of the CFU colony formation experiment. CFU-GM, colony-formation unit of granulocyte-monocyte; CFU-G, colony-formation unit of granulocyte; CFU-GEMM, pluripotent progenitor cells; N = 3.

In order to test this hypothesis, we performed the colony formation assays to identify the natural differentiation ability of HSCs and the lineage and monophyletic differentiation under the action of cytokines. Purified HSCs (Lineage^-^cKit^+^Sca1^-^) were extracted from mouse bone marrow by immunomagnetic bead sorting method. G-CSF, rLZ-8, or equal volume of medium was added to the semi-solid medium. The number of cell colonies was counted after culture for 9 days, and the lineages regulated by cytokines were determined according to the characteristics of the individual cell colony (**Figure 5A**). The results showed that a large number of HSCs were differentiated into CFU-GEMM (44.74%) colonies under the action of high concentration of rLZ-8 (20 μg/ml) and into CFU-GM (58.25%) and CFU-G (38.83%) in the presence of low concentration of rLZ-8 (5 μg/ml), while G-SCF stimulated the HSCs for differentiation into CFU-GM (17.95%) and CFU-G (82.50%) (**Figure 5B**). These findings indicated that rLZ-8 acts on HSCs and stimulates their differentiation into GMPs.

### rLZ-8 Induced the Mobilization of HSCs by Upregulating the Expression of CXCR4 and Downregulating the Expression of SDF-1 in Intramedullary Cells

**Figure 6:**
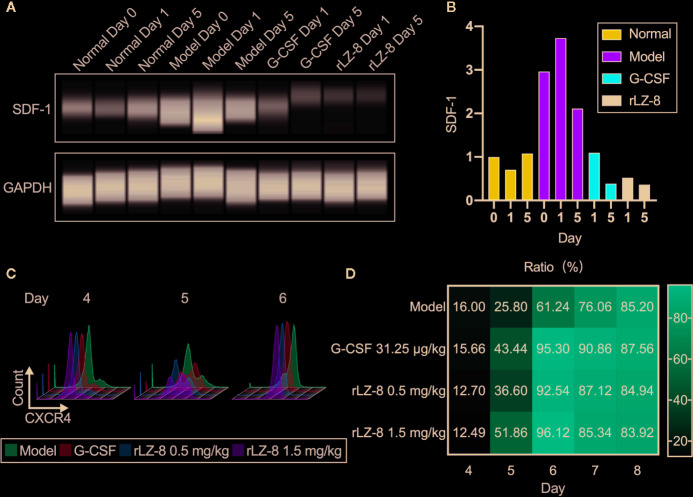
rLZ-8 upregulated the expression of CXCR4 and downregulated the expression of SDF-1 in BMCs. **(A)** Wes detection of SDF-1 protein expression in bone marrow supernatant on days 0/1/5. Normal: Vehicle, G-CSF: 31.25 μg/kg, rLZ-8: 1.5 mg/kg. **(B)** SDF-1 density of each band from **(A)** was measured and compared to the normal group (Day 0). **(C)** FCM detection of CXCR4^+^ cell population in the bone marrow of mice in different treatment groups on experimental days 4/5/6. Model: Vehicle, G-CSF: 31.25 μg/kg, rLZ-8: 0.5 mg/kg, rLZ-8: 1.5 mg/kg, N=10. **(D)** Analysis of CXCR4^+^ cell population ratio in the bone marrow in different treatment groups, N=10.

G-CSF induced the mobilization of HSCs by decreasing the intramedullary expression of SDF-1 protein and increasing the expression of its receptor CXCR4. Similar results were obtained with rLZ-8. Western blot assay confirmed that intramedullary SDF-1 was significantly elevated after the intraperitoneal injection of cyclophosphamide (**Figure 6A**). The intramedullary expression of SDF-1 was significantly reduced at 24 h after the single injection of G-CSF or rLZ-8 as compared to that in the model group. After 5 days of continuous injection of G-CSF or rLZ-8, the intramedullary level of SDF-1 could be barely detected. Consecutively, the SDF-1 content in the model group was still higher than that in the normal group (**Figure 6B**). Moreover, we also detected the CXCR4^+^ cells in the bone marrow of mice in each group on D4–8 of the experiment (**Figure 6C**) that differed significantly as compared to the model group. On D5 of the experiment, the intramedullary CXCR4^+^ cell population in the G-CSF group (43.440 ± 4.554%), the rLZ-8 0.5 mg/kg treatment group (36.600 ± 1.243%), and the rLZ-8 1.5 mg/kg treatment group (51.860 ± 5.946%) showed a significant increase as compared to that in the model group (25.800 ± 2.508%) (**Figure 6D**). These results indicated that similar to G-CSF, rLZ-8 caused a significant decrease in the level of intramedullary SDF-1 protein in the model mice and promoted the expression of CXCR4 in the intramedullary cells. Moreover, the mobilization of HSCs was induced by regulating the intramedullary CXCR4-SDF1 metabolic pathways. In addition, CXCR4 and its ligand SDF-1 played critical roles in regulating the transport of neutrophils. Also, the intramedullary neutrophils in the model group increased but were not released into the peripheral blood, indicating that rLZ-8 promoted the release of neutrophils from the bone marrow to the peripheral blood.

### Binding of rLZ-8 to CSF1R Might Promote the Activation and Differentiation of HSCs

**Figure 7:**
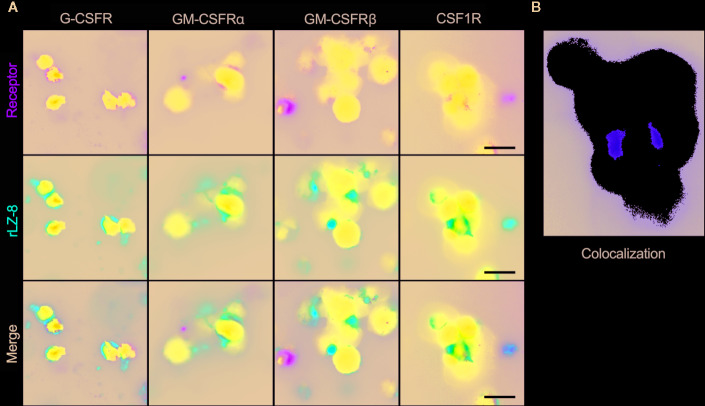
rLZ-8 exhibited fluorescence colocalization with CSF1R in HSCs. **(A)** Position of G-CSFR/GM-CSFRα/GM-CSFRβ/CSF1R (green) and rLZ-8 (red) immunofluorescence on the surface of HSCs, bar=10 nm. **(B)** Analysis of fluorescence colocalization position of CSF1R and rLZ-8 (yellow) with ImageJ in the box of panel A.

In order to determine the activity of rLZ-8 on HSCs, four classes of lineage hemocyte growth factor receptors in the hematopoietic system, G-CSFR, GM-CSFRα, G-CSFRβ, and CSF1R, on the surface of enriched Lineage^-^cKit^+^Sca1^-^ cells were marked by immunofluorescent colocalization experiments. Simultaneously, fluorescent-labeled rLZ-8 was added, and the fluorescence colocalization with the corresponding receptors was observed using SIM in order to judge the receptor of rLZ-8 on the surface of HSCs. The results showed that the four types of receptors were expressed on the surface of HSCs and distributed in various regions. The localization of fluorescent rLZ-8 was not coincident with the fluorescent-tagged positions of G-CSFR, GM-CSFRα, and GM-CSFRβ, which were judged as non-rLZ-8 receptors (**Figure 7A**). The fluorescent region of CSF1R and rLZ-8 with an extremely high rate of overlap were designated as the receptors of rLZ-8 (**Figure 7B**).

## Discussion

The development of biotechnology has emphasized the various pharmacological functions of fungal immunomodulatory protein (FIP) in the discovery and development of new drugs. Since the first FIP (LZ-8) was isolated and purified in 1989, 10 FIPs have been identified, including FIP-gts (G. tsugae), FIP-gja (G. japoncium), FIP-gmi (G. mic), FIP-fve (Flammu linavelutipes), and FIP-vvo (Volvariella volvacea) (Li et al., 2014). The genetic structure and function of FIPs from different fungi have been elucidated and great similarities have been detected with the immunoglobulin superfamily. This characteristic played a major role in anti-tumor activity, anti-allergic activity, promotion of lymphocyte proliferation, induction of cytokine expression, and anti-graft rejection (Li et al., 2011). Although sequence similarities were observed between family members of FIPs, the differences in the functions could not be ignored. Firstly, the difference in surface hydrophobicity of proteins altered the FIP structure and exhibited different protein activities. Secondly, the difference in electrostatic potential formed a unique structure, which affected the interaction of two homologous monomers. FIPs were suitable for *in vitro* expression and structural modification owing to their low molecular weight (13 kDa, 110–114 amino acids) (Li et al., 2018). Thus, these could be utilized for the industrial production of pharmaceutical molecules using genetically engineered strains to obtain high-performance proteins. Therefore, the related research of FIP can be developed further with unlimited mining potential.

An interesting finding from this work is that rLZ-8 promotes the self-renewal of HSCs and their differentiation into granulocyte-derived progenitor cells. The ratio of the neutrophil population is continuously increasing, which is a process of continuously replenishing cells. However, the ratio increases after G-CSF treatment, followed by a decrease and again by an increase (**Figure 3**). This raises the question, what causes this therapeutic difference between the G-CSF and the rLZ-8 group? Both G-CSF and rLZ-8 promote the release of neutrophils through CXCR4-SDF1 axis. So, the difference appears to be due to the different avenues of progress in the differentiation of HSCs. G-CSF acts on granulocyte progenitor cells expressing G-CSFR, but it does not induce the proliferation of all HSCs, and G-CSF-mobilised HSCs have lower repopulating potential per cell than untreated HSCs, contributing to their ultimate exhaustion (Patterson and Pelus, 2016). In addition, as the CFU result confirmed, the concentration of rLZ-8 is important for the realisation of different directions of differentiation. Furthermore, cytokines in the microenvironment play an important role in the differentiation (Zhang and Lodish, 2008). A new current view of how HSCs respond to an environmental challenge and implicate stress-induced cytokines is that lineage-specific cytokines can act directly on HSCs *in vitro* and *in vivo* to instruct a change of cell identity (Mossadegh-Keller et al., 2013). For example, CLPs isolated from mice transgenic for interleukin 2 receptor-β (IL-2Rβ) differentiate into macrophages and granulocytes *in vitro* when stimulated with IL-2 (Kondo et al., 2000), and rLZ-8 can induce IL-2 gene expression (Lin et al., 2009). In a subsequent study, we plan to further improve the *in vivo* model to study the effect of rLZ-8 or G-CSF on the microenvironment related to hematopoietic stem cell differentiation.

Additionally, the colony-stimulating factor 1 receptor (CSF1R) was a class III receptor tyrosine kinase (RTKIII) with a highly glycosylated extracellular region, consisting of five immunoglobulin domains (D1–D5), a transmembrane domain, and an intracellular domain (Felix et al., 2015). Also, it is a receptor of M-CSF, another important member of the CSF family. In the hematopoietic system, CSF1R was expressed in relatively primitive multipotent hematopoietic cells, mononuclear phagocytic progenitor cells, monocytes, and tissue macrophages. It is the only kinase that could be activated by two different cytokines: CSF-1 and IL-34. Both bound to the extracellular domain of CSF-1R to trigger the receptor dimerization and intracellular autophosphorylation, which in turn, initiated the intracellular signal transduction. Different ligand recognition patterns and different binding sites led to differential dimerization and activation of the receptor (Chihara et al., 2010; Felix et al., 2013; Stanley and Chitu, 2014). rLZ-8, as another putative ligand of CSF1R, with different binding sites for CSF-1 and IL-34, was the underlying factor for diverse dimerization. This phenomenon further affected the activation of downstream metabolic pathway and resulted in diversified self-renewal and differentiation of HSCs. Therefore, exploring the binding sites of rLZ-8 with CSF1R and the activation mode of downstream intracellular signaling pathways would complement the detailed molecular mechanism of immunoregulation of the FIP protein. Also, the development and utilization of such proteins derived from medicinal plants need further investigation.

In conclusion, the present study showed that rLZ-8 promote the differentiation of HSCs into granulocyte cell line by binding to CSF1R. It could also promote the release of mature neutrophils from bone marrow to peripheral blood by upregulating the expression of CXCR4 and downregulating the expression of SDF-1 to achieve the treatment of neutropenia by cyclophosphamide in mice. These results preliminarily demonstrated the mechanism of *Ganoderma lucidum* immunoregulatory protein in the treatment of chemotherapy-induced neutropenia, indicating its potential to act as a substitute or supplement for the prevention of neutropenia during tumor chemotherapy.

## Data Availability Statement

All datasets generated for this study are included in the article/**Supplementary Material**.

## Ethics Statement

The animal study was reviewed and approved by the Animal Welfare and Research Ethics Committee at Jilin University in accordance with the animal welfare laws and the regulations of the Association for Assessment and Accreditation of Laboratory Animal Care (AAALAC).

## Author Contributions

CL and XL contributed to the conception and design of the study. XL, WG, and XY carried out the experiments. XS and WH performed the statistical analysis. CZ analysed the FCM data. XZ provided us the rLZ-8 protein standard. XL wrote the first draft of the manuscript. HZ and FS wrote some sections of the manuscript. All authors contributed to the article and approved the submitted version.

## Funding

This study was financially supported by the Science and Technology Project Foundation of Jilin Province (No.20190304035YY, 20170311056YY, 20150204048YY).

## Conflict of Interest

Author XZ was employed by Changchun Intellicrown Pharmaceutical Co. LTD.

The remaining authors declare that the research was conducted in the absence of any commercial or financial relationships that could be construed as a potential conflict of interest.

## Abbreviations

LINGZHI-8, LZ-8, *Ganoderma lucidum* immunoregulatory protein; rLZ-8, recombinant *Ganoderma lucidum* immunoregulatory protein; G-CSF, Granulocyte-colony stimulating factor; HSCs, Hematopoietic stem cells; CFU, Colony-forming unit; SDF-1, Stromal cell-derived factor 1; CXCR4, C-X-C chemokine receptor type 4; CSF1R, Colony stimulating factor 1 receptor; NF-κB, Nuclear factor kappa-light-chain-enhancer of activated B cells; MAPK, Mitogen-activated protein kinase; EGFR, Epidermal growth factor receptor; WBCs, White blood cells; RBCs, red blood cells; CMPs, Myeloid progenitor cells; GMPs, Granulocyte and macrophage progenitor cells; CFU-GEMM, Colonies containing red blood cells and bone marrow cells; CFU-G, Myeloid colonies with pure granulocyte colonies; CFU-GM, Colonies containing granulosa cells and monocytes; NEUT, Neutrophil; FIP, Fungal immunomodulatory protein.
