# Supplementary material for: Recombinant Ganoderma lucidum Immunomodulatory Protein Improves the Treatment for Chemotherapy-Induced Neutropenia
Source: Front Pharmacol. 2020 Jun 26;11:956. doi: 10.3389/fphar.2020.00956 (PMC7333219; doi:10.3389/fphar.2020.00956)
Supplement: Supplementary file 1 [file DataSheet_1.pdf]

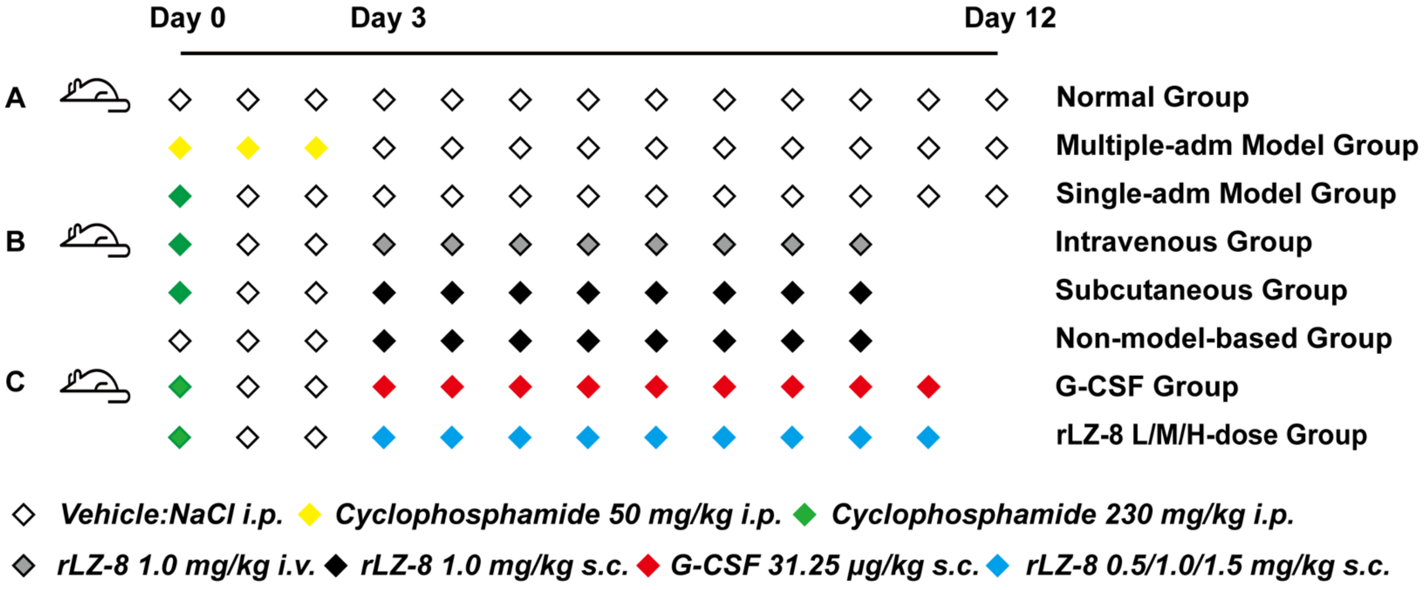

Supplementary Figure 1. Method of establishing CIN mouse models and different treatment plans of G-CSF or rLZ-8 for comparing the effects on WBC count.

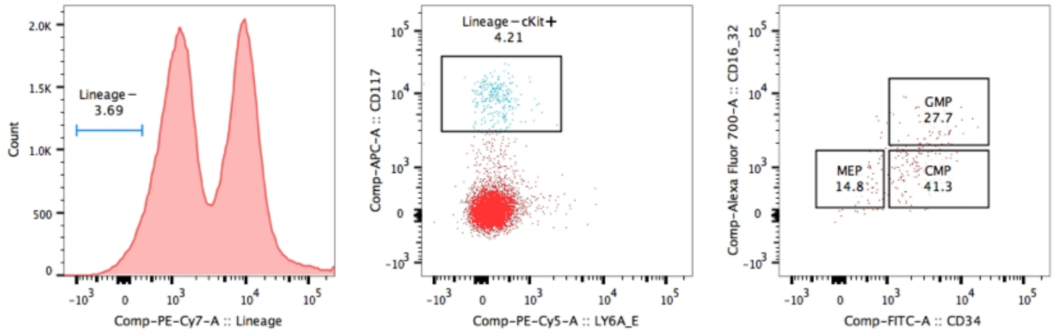

Supplementary Figure 2. BM from mice was assayed by multiparameter FACS for active proportion of primitive HSC populations. BM was stained with antibodies against Lineage markers, CD117, LY6A/E, CD34, CD16/32, and gated as shown in S Figure 2. At least five mice per group were compared. The proportion of BM corresponding to the HSC. More differentitated progenitors gated in the $Lin^{-}cKit^{+}Sca1^{-}$ population were subsectioned based on CD16/32 and CD34 expression to compare CMP and GMP progenitors.
